# Compact Assessment of Molecular Surface Complementarities Enhances Neural Network-Aided Prediction of Key Binding Residues

**DOI:** 10.1021/acs.jcim.4c02286

**Published:** 2025-02-21

**Authors:** Greta Grassmann, Lorenzo Di Rienzo, Giancarlo Ruocco, Mattia Miotto, Edoardo Milanetti

**Affiliations:** † Department of Biochemical Sciences “Alessandro Rossi Fanelli”, Sapienza University of Rome, P.Le A. Moro 5, Rome 00185, Italy; ‡ Center for Life Nano & Neuro Science, Istituto Italiano di Tecnologia, Viale Regina Elena 291, Rome 00161, Italy; § Department of Physics, Sapienza University, Piazzale Aldo Moro 5, Rome 00185, Italy

## Abstract

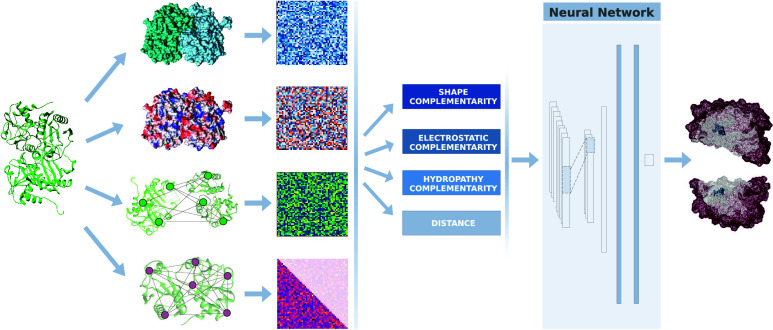

Predicting interactions between proteins is fundamental for understanding the mechanisms underlying cellular processes, since protein–protein complexes are crucial in physiological conditions but also in many diseases, for example by seeding aggregates formation. Despite the many advancements made so far, the performance of docking protocols is deeply dependent on their capability to identify binding regions. From this, the importance of developing low-cost and computationally efficient methods in this field. We present an integrated novel protocol mainly based on compact modeling of protein surface patches via sets of orthogonal polynomials to identify regions of high shape/electrostatic complementarity. By incorporating both hydrophilic and hydrophobic contributions, we define new binding matrices, which serve as effective inputs for training a neural network. In this work, we propose a new Neural Network (NN)-based architecture, Core Interacting Residues Network (CIRNet), which achieves a performance in terms of Area Under the Receiver Operating Characteristic Curve (ROC AUC) of approximately 0.87 in identifying pairs of core interacting residues on a balanced data set. In a blind search for core interacting residues, CIRNet distinguishes them from random decoys with an ROC AUC of 0.72. We test this protocol to enhance docking algorithms by filtering the proposed poses, addressing one of the still open problems in computational biology. Notably, when applied to the top ten models from three widely used docking servers, CIRNet improves docking outcomes, significantly reducing the average RMSD between the selected poses and the native state. Compared to another state-of-the-art tool for rescaling docking poses, CIRNet more efficiently identified the worst poses generated by the three docking servers under consideration and achieved superior rescaling performance in two cases.

## 1 Introduction

Over 80% of proteins operate in molecular complexes,^60^ making the comprehension of protein complex formation fundamental for unveiling physiological cellular processes. Understanding how proteins interact also has significant implications for therapeutic applications, allowing the investigation of the processes leading to pathological conditions. For example, many neurodegenerative diseases are associated with protein aggregates that are seeded by protein–protein binding: developing an effective method for predicting complex formation is crucial for understanding this class of pathologies.^24^

While experimental methods like X-ray crystallography and nuclear magnetic resonance (NMR) spectroscopy enable the large-scale detection of protein interactions, these techniques are often costly and time-consuming.^54^ Moreover, protein–protein interactions have to be precisely and finely tuned,^66^ because binding partners must find themselves among thousands of other types of molecules in the cell.^15,16,46,47,88^ Accurate prediction and understanding of complex formation require unveiling all components of the binding mechanisms, highlighting the importance of computational methods in this domain.^22^ The introduction of AlphaFold2^30^ in 2021 revolutionized the field by providing three-dimensional (3D) structures of proteins (with a resolution close to the experimental data) based solely on their amino acid sequences. This development has significantly enhanced the role of structural information in protein interaction modeling and molecular docking.

Docking tools generate thousands of potential binding poses for two independent structures, helping to address the scarcity of high-quality experimental data on molecular complexes. For example, Yin et al.^80^ recently leveraged data from molecular docking to devise a network for the prediction of native-like docking poses between proteins and ligands. Their approach achieved an average improvement of approximately 27% compared to nine leading deep learning-based methods for bioactivity prediction.

Nonetheless, the identification of near-native models from the large pool of generated models is still a challenge for these techniques. Competitions that evaluate the latest protein–protein docking algorithms, such as CAPRI,^28^ continue to seek effective methods for interface and pose prediction. This is largely due to the absence of robust scoring functions capable of consistently identifying the model closest to the native structure.

Docking tools can be classified as direct or template-based. Template-based methods use homology modeling to predict the complex structure by identifying common patterns through multiple sequence alignments (MSA), given that interacting pairs with over 30% sequence identity often interact similarly.^2^ One of the latest template-based protocols is AlphaFold3, introduced by Abramson et al.^1^ AlphaFold3 can predict complexes including proteins, nucleic acids, small molecules, ions, and modified residues. Thanks to a diffusion-based architecture, it reaches a higher accuracy compared to previous methods.^1,17,79^ However, the availability of homologous proteins is not always guaranteed. Direct docking methods, which search for the complex structure that minimizes free energy within the conformational space, are not constrained by the availability of homologous proteins. On the other hand, these methods require the definition of a computationally feasible free-energy evaluation model and effective minimization algorithms.^69^

Several methods have been proposed to identify the near-native conformations from the large pool of docking-generated models. They can rely on knowledge-based statistical potentials (such as GOAP^85^), conservation of inter-residue contacts (such as CONSRANK^57^) or machine learning methods (e.g., iScore,^20^ TRScore,^25^ MetaScore,^31^ DOVE,^76^ DeepRank,^63^ GNN-DOVE,^75^ and DeepRank-GNN^61^). The latter have shown the best results,^77^ as they can discover complex nonlinear combinations of features.

Indeed, protein interactions are influenced by a variety of factors, including van der Waals forces, electrostatic interactions, hydrogen bonding, hydrophobic effects, and solvent interactions.^10,48,51,55,67,70^ Binding sites exhibit a combination of geometric and chemical complementarities, which determine complex formation specificity and binding stability.^12,13,18,52,53^ However, incorporating more features increases computational demands. Given the already substantial cost due to numerous possible contact patches, developing efficient methods that focus on key interaction features is essential.

One well-known characteristic of binding interfaces is shape complementarity, which is determined by side-chain rearrangement minimizing van der Waals interactions.^10^ Various techniques have been proposed for the evaluation of shape complementarity.^9,19,33,72,87^ In 2021, we proposed a method^44^ based on Zernike polynomials for the description of the shape of surface portions. This approach, which does not require structural alignment between interacting molecules, leverages the rotational invariance of Zernike polynomials for quick evaluation of patches compatibility (in terms of Euclidean distance between Zernike vectors) without considering their mutual orientation,^5,23,45,49^ and blindly identifies true interacting regions in approximately 60% of the cases.^44,50^

More recently, we extended the same formalism to another property that can be described with numerical values assigned to each surface point: the electrostatic potential.^21^ We demonstrated that interfaces also exhibit electrostatic complementarity. To evaluate it, we applied the Zernike method to molecular surfaces for which the electrostatic potential was calculated with the Poisson–Boltzmann equation. This protocol discriminates between transient and permanent protein complexes with an Area Under the Receiver Operating Characteristic Curve (ROC AUC) of approximately 0.8.

Another fundamental contribution to protein binding is the degree of hydrophilicity and hydrophobicity at the interfaces: interacting sites must present a balance between the former, ensuring the structural stability of each partner, and the latter, which is needed to ensure binding. In previous works, we developed a hydrophobicity scale that assigns a hydrophobicity index *H* to each amino acid based on changes observed in the hydrogen bond network of water molecules surrounding each given compound during Molecular Dynamics (MD) simulations.^11^

Here, taking advantage of the speed and compactness of the biophysical characterization of interacting regions in protein complex structures through the Zernike polynomial-based formalism, we present a novel method to evaluate shape, electrostatic, and hydropathy complementarity through a unified computational protocol. By characterizing the radial variability of these aspects at interfaces, we find that they are maximized within an area of 10 Å radius. More specifically, we define new binding matrices that compactly describe shape, electrostatic, and hydropathy complementarity between surface patches of this size. These matrices are used to train a Neural Network (NN) to identify interacting residues at the core of binding sites.

The proposed Core Interacting Residues Network (CIRNet), was able to successfully identify core interacting residues both on a balanced data set of 1086 complexes and in a blind-search on a second data set of 100 complexes. Core interacting residues are defined as pairs of residues closer than 3 Å and where both have a distance from the centroid of the interface smaller than 5 Å.

To evaluate CIRNet’s performance, we used its predictions as additional information for selecting the most reliable models from three publicly available docking servers: ClusPro,^8^ PyDock,^6^ and LZerD.^7^

When tested on 30 complexes, we observed that the docking scores did not always reflect the similarity to the native complex. Using the CIRNet prediction as an additional filter for the docking poses, we were able to extract some of the worst poses among the first ten proposed by ClusPro, PyDock, and LZerD.

Our results show that the NN predictions for core interacting pairs of poses that strongly differ from the native structure are usually lower. Thus, by removing poses whose core interacting residues are not recognized as such by CIRNet, we can partially filter out structures that despite being highly ranked are far from the native pose. Even when the true conformation does not present core interacting residues, CIRNet can still identify pairs that are proposed as core interacting residues by the docking but do not meet the physical and geometrical criteria for core binding regions.

To offer a comparison with a state-of-the-art tool for the rescaling of the docking poses, we applied to the same data set both CIRNet and TRScore.^25^ By comparing the RMSD of the poses proposed by the three docking servers and the score assigned to each of them by CIRNet and TRScore, we observed that our protocol demonstrated better or comparable performance in removing the worst docking poses. For two out of the three docking servers, CIRNet also showed better performance as a ranking methods, as shown by a better correlation between the predicted score and the RMSD.

## 2 Results and Discussions

### 2.1 Binding Regions Have a Hydrophobic Core and a Charged Rim

To perform a quantitative wide-ranging spatial characterization of the interfaces we aimed to predict, we selected from the complexes proposed by Gainza et al.^19^ the same balanced data set already used by Milanetti et al.,^44^ counting 3721 complexes with available structural data. The data set, named “CIRNet data set”, includes 1730 homodimers, which can be categorized into 614 dimers with an Identical Binding Region (IBR-hom), 998 Shifted Binding Region (SBR-hom), and 118 non-Identical Binding Region (nIBR-hom), based on the similarity of the interacting patches. Additionally, the data set contains 1991 heterodimers (Het). See ref (21). and Methods for more details.

For each complex, we computed the solvent-accessible surface (as described in the Section 4) and evaluated the chemical features of the surface regions surrounding the geometrical center of the interfaces, divided into annular regions with a 1 Å radius. We defined the interfaces as the “hot spots” on a protein surface within 6 Å of its partner surface. Given that the interfaces in the “CIRNet data set” have an average maximum radius of 17 Å, we analyzed the contributions of amino acids within a 20 Å radius from the center of the interfaces.

**Figure 1:**
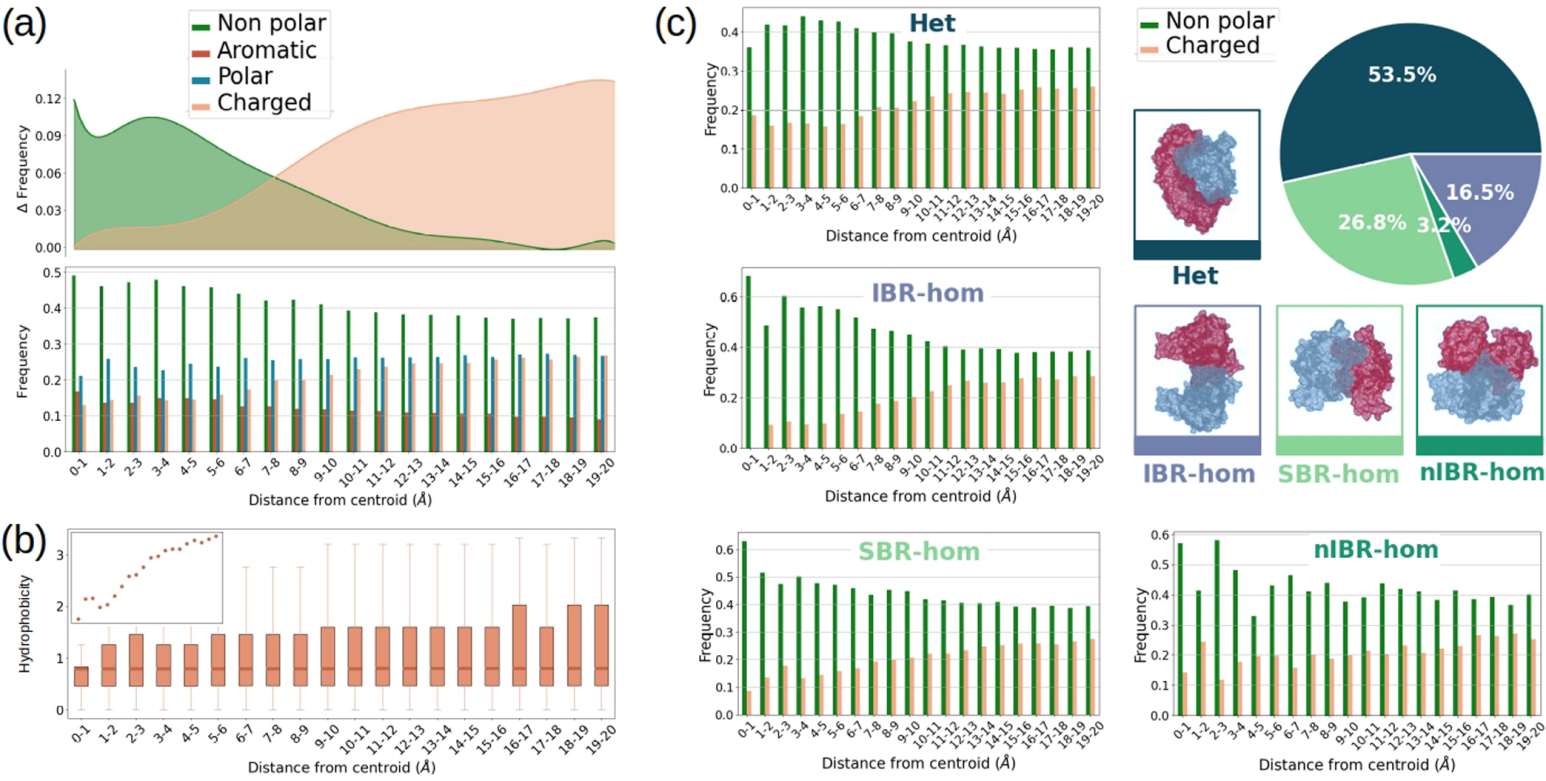
Amino acid composition, hydropathy properties, and dimer classification of the “CIRNet data set” interfaces. (a) On the bottom the frequency of each of the four classes of amino acids (nonpolar, aromatic, polar, and charged, represented in green, red, blue, and yellow respectively) in 1 Å-wide annular regions at increasingly larger distances from the center of the binding sites. For nonpolar (i.e., amino acids characterized by a higher hydrophobicity) and charged residues, the difference between the frequency value in each annular region and the minimum frequency of that class is shown on top. (b) The box plot displays the hydrophobicity (*H*) values of the residues included in the annular regions defined in (a). The inset shows the mean *H* value for each annular region. (c) The “CIRNet data set” includes heterodimers (Het) and homodimers with identical (IBR-hom), nonidentical (nIBR-hom), and shifted binding regions (SBR-hom), as shown by the percentages in the pie plot. An example for each class is provided in the four inserts. The frequencies of nonpolar and charged residues shown in (a) are divided according to these classes.

It has already been discussed how at the interfaces, compared to the rest of the surface, polar and charged residues are less frequent while hydrophobic amino acids are more present.^29,68,78^ Here, we report a quantitative analysis of the radial distribution of amino acids near the core of interacting regions. As shown in Figure 1a hydrophobic residues are particularly predominant at the core of the binding regions compared to the other classes, in accordance with what is already known.^62^ Up to a distance of 10 Å from the interface centroid, they oscillate between approximately 50 and 40% of the total. We define this region as the core of the binding site. At the rim of the binding sites, hydrophobic residues become less abundant, although they remain the predominant class with a frequency of about 37%. The opposite trend is observed for charged residues: at the core of the binding site, they account for less than 20% of the total residues, while at the rim, their frequency increases by one-fourth.

Figure 1b shows that core residues tend to have a higher degree of hydrophobicity (indicated by the descriptor *H*). Indeed, residues can establish strong hydrophobic interactions when they have low hydrophobicity values, as defined by the scale proposed by Di Rienzo et al.^11^

Figure 1c shows how the core and rim regions of the interfaces have similar trends across all four dimer classes. In all cases, nonpolar residues are particularly predominant at the core, while the frequency of charged amino acids increases outside a 10 Å radius. Nonpolar residues are more frequent at the interfaces of homodimers than heterodimers; for homodimers, shape complementarity seems to be a less stringent requirement for binding.

### 2.2 Binding Sites as the Regions were Shape and Hydropathy Complementarity are Maximized

**Figure 2:**
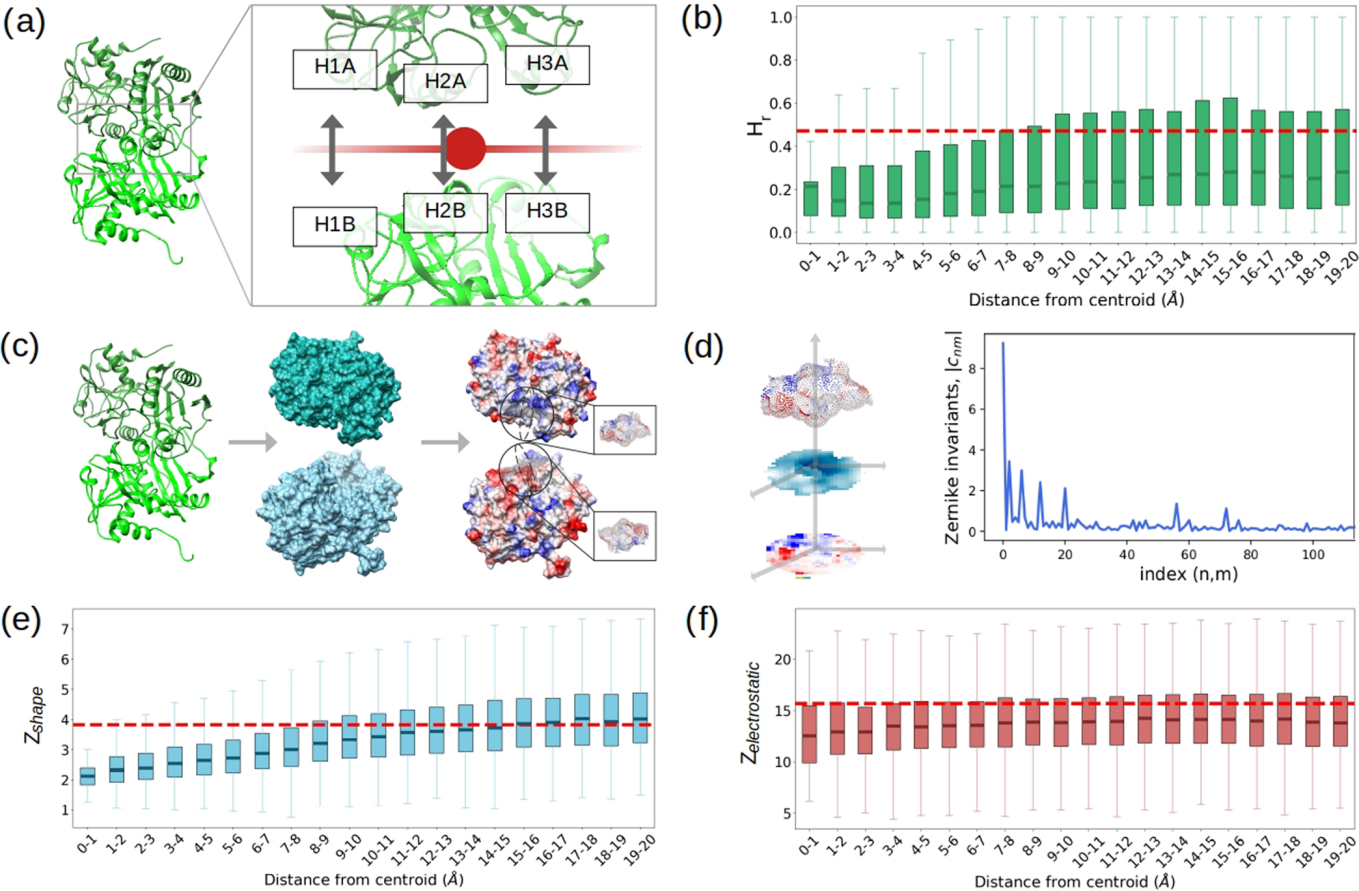
Characterization of complementarities at the interfaces. (a) The center of each binding site is identified. Starting from the hydrophobic (*H*) index of the interacting residues facing each other (closer than 3 Å), the hydropathy complementarity (*H_r_*) for each residue pair is computed. (b) Box-plot of the *H_r_* values of interacting residue pairs in 1 Å-wide annular regions at increasingly larger distances from the center of the interfaces. The dotted red line indicates the mean *H_r_* value obtained between random noninteracting pairs. (c) Molecular representations of two proteins forming a dimer. The solvent-exposed and electrostatic potential surfaces are obtained from the secondary structure. In the latter, each point is colored according to its electrostatic potential value. Spheres with a 9 Å radius are used to select a patch on the surface: in the zoomed portion, the selected points. (d) Two-dimensional (2D) projections of one of the patches selected in (c). In the blue scale, the shape projection: the color of each pixel is determined by the distance *r* from a predefined origin (see Section 4 for details) of the surface points projected inside of it. In the blue-red scale, the electrostatic projection: colors are determined by the electrostatic potential values of the projected points. Each projection is a 2D function that can be associated with a Zernike vector. The Euclidean distances *Z***_*s*_** and *Z*_el_ between the shape or electrostatic vectors associated with two patches quantify their complementarities. (e) Box-plot of the Euclidean distances *Z*_s_ between the Zernike vectors describing the shape of interacting patches in 1 Å-wide annular regions at increasingly larger distances from the center of the interfaces. The dotted red line indicates the mean *Z*_s_ value obtained between random noninteracting patches. (f) Same as in (e), but for *Z*_el_, measuring electrostatic complementarity.

To further characterize the hydrophobic interactions at the interfaces, we compared the *H* value of residues facing each other and evaluated the strength of these interactions at increasing distances from the center of the interfaces (see Figure 2a), thus allowing the evaluation of a hydropathic complementarity. This analysis increases the complexity of investigating the nature of protein binding, as we are not only examining the distribution of the degree of hydrophobicity of the residues forming the interfaces (as previously reported) but also measuring the local matching of hydropathy in each region of the interfaces of protein–protein complexes.

As known, the bond between amino acids mainly depends on their hydrophobic or hydrophilic nature. Interactions between hydrophobic residues (low *H* values) are strong because such bonds are energetically favorable, reducing the exposure of hydrophobic surfaces to the aqueous environment. Strong interactions occur between hydrophilic residues (high *H* values) as well, since they can form hydrogen bonds. However, when a hydrophobic residue interacts with a hydrophilic residue, there is no tendency for a strong interaction.

Given these interaction tendencies, we can model the bond strength between two residues with their *H* indices. To quantify this strength, we defined the hydropathy complementarity between two residues *A* and *B* as a parabolic function
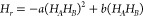
1where *H*_*A*_ and *H*_*B*_ are the hydrophobic indices of the two amino acids, and the parameters *a* and *b* are set to 0.033 and 0.363 respectively. *H*_*r*_ reflects the idea that residues with similar hydrophobicity values (both high or low) have stronger interactions compared to those between residues with opposing hydropathy characteristics. Values close to zero correspond to strong interactions: the closer *H*_*r*_ is to one, the weaker the bound between the residues. Interface residues tend to be hydrophobic: the mean hydropathy complementarity computed from the *H* indices of randomly extracted noninteracting residues is *H*_*r*_ = 0.47, as shown by the red dotted line of Figure 2b. Close to the interface centroid (residues closer than 4 Å), *H*_*r*_ has the lowest value. The rest of the core region shows a slow increase of *H*_*r*_, which then reaches and maintains a stable value at the rim. Almost all hydropathy complementarity values calculated for protein–protein interfaces are lower than the mean *H*_*r*_ value computed from the *H* indices of random residues extracted from the nonbinding regions of two interacting proteins. This is especially true in the central regions of the interfaces. This marked distinction in hydropathy complementarity between interacting and noninteracting patches highlights the importance of quantifying this property for a comprehensive characterization of protein–protein binding, thereby enabling the integration of the descriptor into the algorithm for predicting interacting regions.

Since protein binding depends on an interplay between various contributions on the molecular surface, we extended this analysis to two more features: shape and electrostatic complementarities. For the former, it is widely known that the side-chain optimization of the short-ranged van der Waals interactions between interfaces leads to a local shape complementarity of the proteins’ molecular surfaces. The role of electrostatic interactions, including hydrogen bonding, ionic/Coulombic, cation −π, π–π, lone-pair sigma hole, and orthogonal multipolar interactions,^3,11,66^ is more debated:^3,66^ while it is understood that they can bring close distant partners and reorient them,^65^ the role they play in binding on small distances is still uncertain.^37,71,81,84,86^ Recently, we characterized the role of these interactions in protein binding in surface regions with a radius of 9 Å, where shape complementarity is maximized^21^ that is, the Lennard–Jones potential energy is minimized. We proposed a computational protocol that can distinguish between interacting and noninteracting regions by describing their electrostatic potential surfaces with vectors and comparing these descriptors. This method is based on a novel computational strategy proposed in 2021 by Milanetti et al.^44^ to quickly evaluate shape complementarity at interfaces using 2D Zernike polynomials.

In a blind search, the Zernike-based protocol can identify binding regions in 60% of cases, making it useful as an initial filter for identifying potential interacting regions with low computational time and cost. The extension of the methodology based on the Zernike formalism to the electrostatic potential surface enabled a compact characterization not only of the shape contribution, which is dominated by the Lennard–Jones potential but also of the Coulomb potential, which, when integrated into the computational procedure, plays a crucial role in discriminating between transient and permanent interactions.^21^

The main steps of the Zernike-based method are shown in Figure 2c,d. Starting from a protein structure, we compute the solvent-exposed surface and the corresponding electrostatic potential surface, where each surface point is described by its spatial coordinates and by the value of the electrostatic potential generated by the protein at that point. See the Section 4 for more details. Next, we define a surface patch by selecting all the points on the electrostatic potential surface contained in a sphere with a 9 Å radius centered on a surface point. This patch is then projected onto a plane so that it can be associated with two 2D matrices. In the first one, each pixel derives from the spatial positions of the points projected inside it. In the second matrix, each pixel is the mean of the electrostatic potential values of the projected points. The series expansion of such matrices (2D images defined on a unit radius) allows transitioning from a 2-dimensional object (matrix) to a 1-dimensional object (vector), which is defined by the invariant coefficients of the Zernike polynomials (see Section 4 for more details). The Euclidean distance between the Zernike vectors describing two patches quantifies their complementarity, in terms of shape or electrostatic.

Figure 2e depicts the Euclidean distances *Z*_s_ between the Zernike vectors describing the shape of the surface patches relative to residues at increasing distances from the interface centroid. Lower *Z*_s_ values indicate higher shape complementarity (i.e., higher binding propensity^44^). The core region is clearly distinguished from the rest of the interface by higher shape complementarity values compared to the mean *Z*_s_ between random noninteracting residues (red dotted line), quantitatively and compactly demonstrating the role of van der Waals interactions at protein interfaces in terms of shape complementarity of the interacting molecular surfaces. This distinction is less marked for electrostatic complementarity, as shown in Figure 2f. The level of electrostatic complementarity seems to be more uniform between core and rim.

However, both regions have lower Euclidean distances *Z*_el_ between the Zernike vectors describing the electrostatic potential of the surface patches compared to that between random noninteracting residues.

These observations allow us to quantitatively define the typical binding site as the region where shape complementarity and hydrophobic contributions are maximized. This region has a radius of approximately 10 Å. Interestingly, this size is consistent with the radius of the patches that optimize the performance of the Zernike protocol, as discussed in previous studies.^21,44^

### 2.3 Interplay between Shape, Electrostatic, and Hydropathy Complementarity Characterizes Core Interacting Residues Pairs

We have discussed how the essential interactions for binding stability are maximized in a core region at the interface with a radius of approximately 10 Å. We have also shown that the Zernike-based method can characterize each region on the protein surface in terms of shape and electrostatic potential, enabling rapid comparison between different patches. Leveraging these results, we introduce CIRNet, a NN architecture that can identify pairs of core interacting residues by combining the characterization of shape, electrostatics, and hydropathy complementarities.

**Figure 3:**
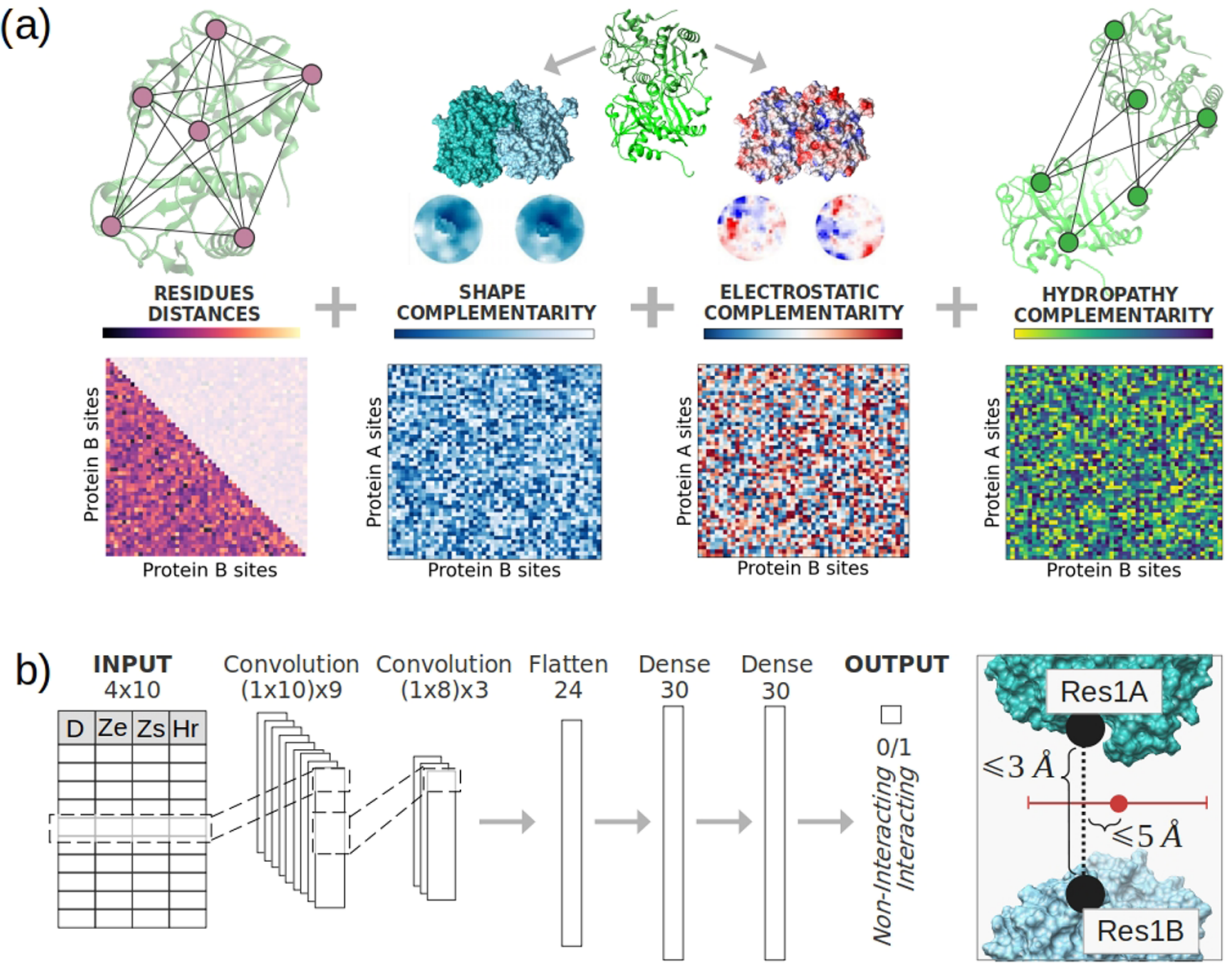
CIRNet architecture. (a) Shape, electrostatic, and hydropathy complementarity (*Z*_s_, *Z*_el_, and *H*_*r*_ respectively) are computed for each possible pair of residues. This process results in three matrices for each protein pair, representing shape, electrostatic, and hydropathy complementarity between all residues (blue, red-blue, and blue-green color scales). For one of the proteins, a matrix with the distances between all of its residues (pink color scale) is also defined. (b) Each residue pair is associated with a 4 × 10 matrix that summarizes the shape, electrostatic, and hydropathy complementarity levels between the first analyzed residue and the neighborhood of the second one. This matrix is fed into a NN with two convolutional layers and two dense layers. The NN then classifies each residue pair based on its probability of being at the core of an interface. Core interacting residues are defined as those closer than 3 Å and within 5 Å from the interface center.

Figure 3a illustrates the procedure leading to the data given as input to CIRNet. Given a pair of proteins, we compute for each possible residue pair *Z*_s_, *Z*_el_, and *H*_*r*_. This results in three binding matrices with sizes *N* × *M*, where *N* and *M* are the number of residues in the first and second proteins, respectively. For the second protein (protein *B*), we also compute the distance between its residues. This fourth matrix identifies the first nine neighbors of each residue in *B*. This number of neighbors defines an area with an average radius of 10 Å, corresponding to the core regions expected to be binding discriminants. For each pair of residues 1*A*, 1*B*, we build a matrix with four columns and ten rows, which is the input of the NN. The first row has the shape, electrostatic, and hydropathy complementarity value between 1*A* and 1*B*. The fourth value is the distance of residues 1*B* from itself (0). The following rows have the complementarities between residue 1*A* and the closest neighbor of 1*B*, 2*B*, and the distance between 2*B* and 1*B*. The second row refers to the second neighbor, and so on.

The resulting matrix is given as input to CIRNet, whose architecture is shown in Figure 3b and described more in-depth in the Section 4. CIRNet assigns to each pair of residues a value reflecting the probability of it being a pair of interacting residues at the core of the interfaces. “True” labels are assigned to the residues that are closer than 3 Å and that have a distance *R* from the centroid of the interface smaller than 5 Å. These parameters resulted in the best training of the network. Table 1 shows the ROC AUC between the distributions of the classification of core interacting pairs and all the others for increasing *R* values. Smaller *R* were not considered to avoid having too few cases of core interacting pairs.

**Table 1:** NN Classification Efficacy Depending on Data Labeling^a^

*R*	ROC AUC
<5 Å	0.78
<6 Å	0.78
<7 Å	0.74
<8 Å	0.76
<9 Å	0.70
<10 Å	0.71
<11 Å	0.70
<12 Å	0.68

a ROC AUC of the NN classification between real interacting and non-interacting residue pairs for increasing maximum distances *R* from the interface center, within which residue pairs are labeled as interacting.

**Figure 4:**
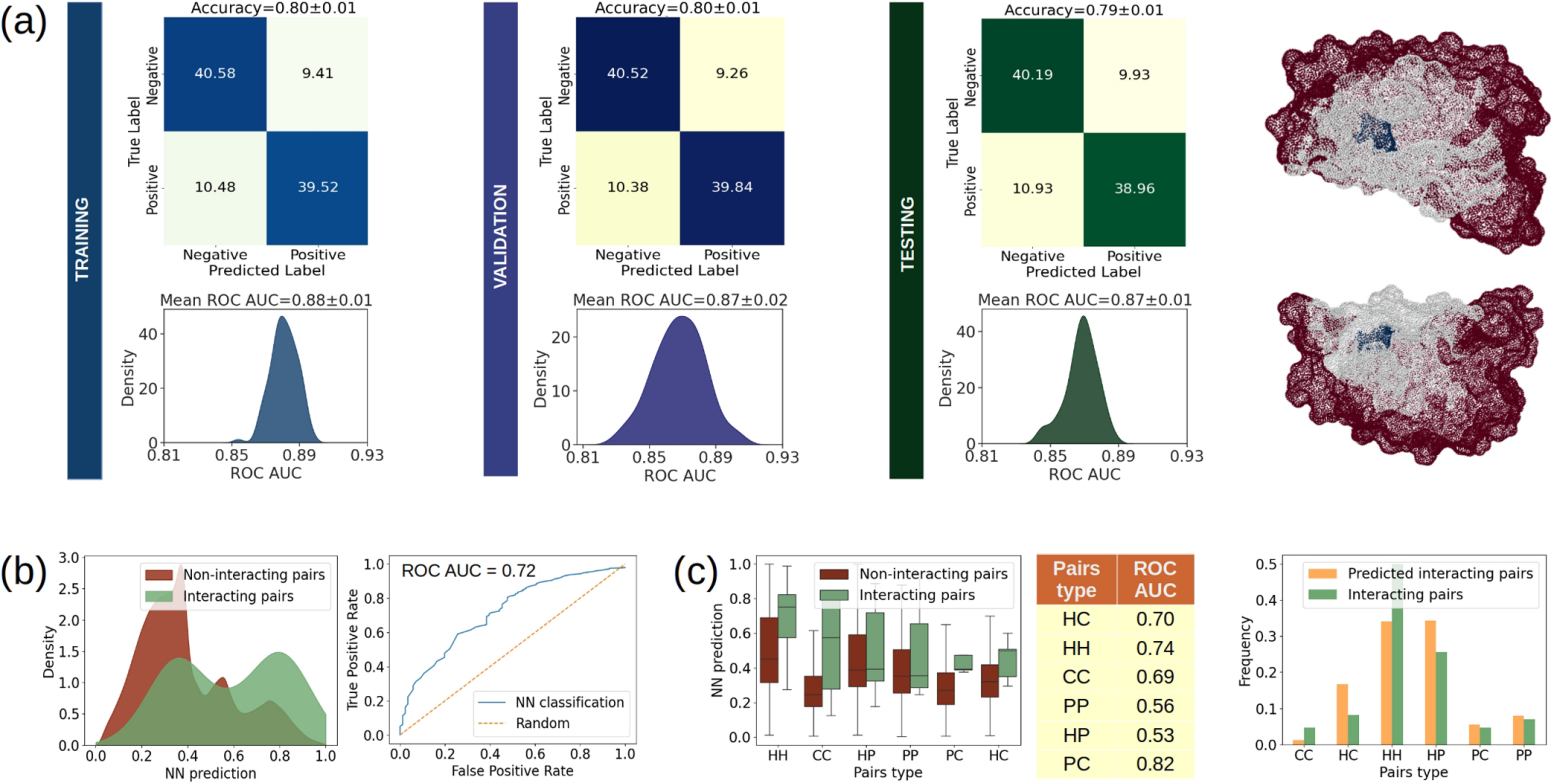
Accuracy of CIRNet during training and testing, and blind-search performance for core interacting residues based on chemical significance. (a) The mean results for 100 repetitions of CIRNet training, validation, and testing are shown in blue, violet, and green, respectively. For each phase, the mean confusion matrix is presented along with the average accuracy. The distributions of ROC AUC values for each repetition are displayed, reflecting the classification performance between core interacting residues and decoys. The mean ROC AUC for these distributions is indicated above each plot. An example complex is provided on the right, showing two interacting residues (in blue) at the core of an interface (in gray) identified by the network as the most interacting. (b) On the left, the CIRNet predictions from a blind-search for core interacting residues across a data set of 100 complexes are displayed. The NN classifications are categorized into true core interacting residues (green) and decoys (red). On the right, the ROC curve between these two distributions is shown, and the AUC value reported. (c) On the left, the distributions from (b) are further stratified based on the chemical nature of the residues. Residue pairs can be hydrophobic–hydrophobic (HH), charged–charged (CC), hydrophobic-polar (HP), polar–polar (PP), polar-charged (PC), or hydrophobic-charged (HC). Pairs are organized according to the mean NN prediction value for core interacting residues. In the table, the ROC AUC for the distributions stratified by residue type. The barplot on the right illustrates the frequency of each residue class among both true core interacting residues and those identified as such by CIRNet.

CIRNet was trained on 2535 complexes, with 20% of this data set used for validation at each epoch. To determine the optimal NN parameters, we created a balanced benchmark with an equal number of core interacting residues and negative examples (i.e., pairs of randomly extracted noninteracting residues). The network was subsequently tested on a balanced set of residue pairs from an additional 1086 complexes. Figure 4a displays the average results from 100 repetitions of the training, validation, and testing procedures. In each repetition, complexes for the three phases were randomly selected from the full “CIRNet data set” described in the Section 4. CIRNet achieved consistent performance metrics across the 100 test repetitions, with a mean accuracy of around 0.79 and a ROC AUC of approximately 0.87. As evidenced by a higher rate of True Negatives relative to True Positives, it showed a better performance in identifying noncore interacting residues compared to core interacting pairs.

To evaluate the robustness of the CIRNet architecture when exposed to novel input data, we tested its performance by training exclusively on homodimers and testing on heterodimers. For this purpose, the CIRNet data set was revisited and divided based on dimer structures. In this case, CIRNet achieved an accuracy of approximately 0.73. While a lower accuracy is expected when training and testing on distinct data types, the observed performance, which is not significantly lower than the accuracy obtained with a mixed data set (∼0.79), suggests that CIRNet demonstrates a degree of robustness to data sets with varying characteristics.

Subsequently, we assessed CIRNet’s ability to blind-test core interacting residues on 100 complexes that were excluded from the training data set and had not been presented to the NN before. In this evaluation, all possible residue pairs were classified by CIRNet, yielding the distribution shown in Figure 4b. The NN-prediction distributions of true noninteracting and interacting pairs are distinguished with a ROC AUC of 0.72, as shown in the right panel of Figure 4b. To further analyze the double-peaked distribution of core interacting residues in the NN prediction, depicted in Figure 4b, we investigated the chemical significance of the network’s learning. Specifically, we stratified the classification of pairs based on residue types (hydrophobic, charged, and polar).

Figure 4c shows that 36% of interacting pairs misclassified by CIRNet are hydrophobic-polar (HP) residues, despite being the second most abundant interacting class (25% of all interacting residues). This is due to the network misclassifying 68% of interacting HP pairs as noninteracting.

The second most frequently misclassified interacting class (28%) consists of hydrophobic–hydrophobic (HH) residues. HH pairs are the most abundant interacting class (50%), yet CIRNet assigns them a score below 0.6 in 28% of cases. HH pairs achieve one of the highest ROC AUC values for distinguishing core interacting residues from negatives, reaching 0.74, comparable to the 0.82 observed for polar-charged (PC) pairs. Hydrophobic-charged (HC) residues make up 14% of misclassified interacting pairs, with prediction scores centered around 0.55, despite accounting for less than 8% of all interacting residues. However, the ROC AUC for HC pairs is one of the highest, at 0.7, indicating a clear distinction between interacting and noninteracting HC pairs.

In contrast, polar–polar (PP) and HP pairs are less clearly distinguished as core interacting residues or decoys, as reflected by their ROC AUC values of 0.56 and 0.53, respectively.

### 2.4 Docking Poses Selection

Having verified CIRNet’s effectiveness in identifying core interacting residues through blind searches, we evaluated its efficiency as a reranking tool for docking results. Docking techniques face the challenge of efficiently ranking hundreds of thousands to millions of possible configurations to identify those closest to the native complex. The need for rapid computation often compromises chemical accuracy, leading to known errors in docking scoring functions.^4,27,56^

We tested CIRNet on three publicly available docking servers: ClusPro, PyDock, and LZerD. ClusPro and PyDock are rigid-body docking methods, while LZerD performs flexible docking. For each server, we docked the individual units of the “Docking data set”—composed of 30 complexes and described more in detail in the Section 4 and selected the top ten models from each. We then computed the Root Mean Square Deviation (RMSD) between each model and the native pose, and identified residues on each partner within 3 and 5 Å from the interface centroid, corresponding to our definition of core interacting residues. CIRNet provided interaction prediction scores for these selected pairs.

**Figure 5:**
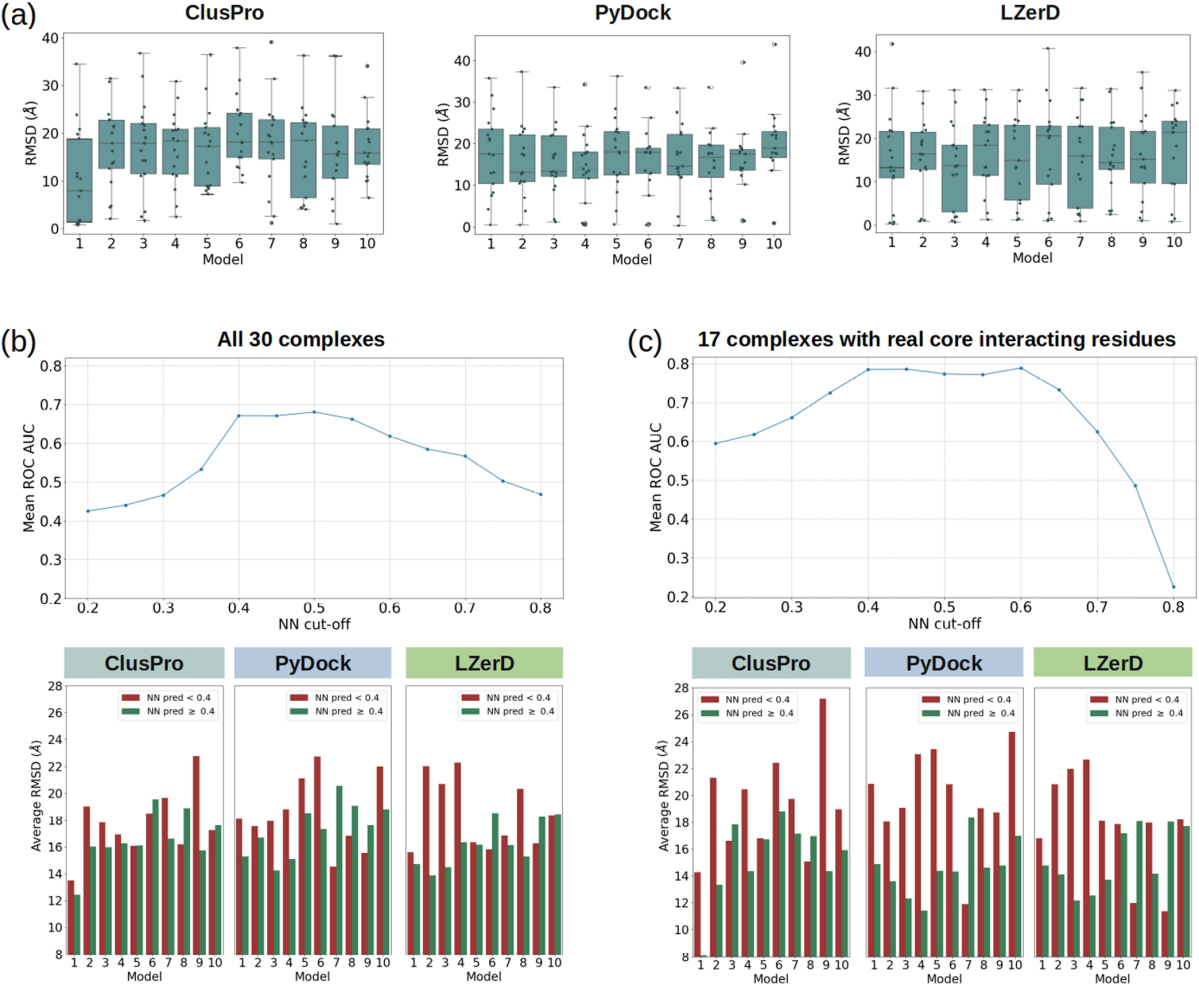
Rescaling of the top ten predicted models of the selected docking servers via CIRNet. (a) The individual units of the “Docking data set” were docked using three publicly available tools: ClusPro, PyDock, and LZerD (from left to right). For each docking server, the RMSD between the first ten models and the native complex is computed. Each subplot reports the RMSD of the docked structures as a function of the docking ranking. (b) For all docked poses, the residue pairs within 3 and 5 Å from the interface centroid are selected. The NN prediction scores are calculated for these pairs and various NN prediction thresholds are applied to classify pairs as core interacting. The top plot shows the difference in RMSD between poses associated with residue pairs classified as core interacting (RMSD_selected_) and those classified as noncore interacting (RMSD_removed_) for increasing NN prediction thresholds. The RMSD is averaged for each of the ten ranks, indicated by the legend. The bottom plot shows the average RMSD of poses selected (green) or removed (red) using an NN prediction threshold of 0.4, averaged across all poses in each ranking level for each docking server. (c) Same as in (b), but only the complexes with core interacting residues in their native pose are considered.

Figure 5a illustrates that docking ranks do not consistently reflect similarity to the native pose. For ClusPro, the median RMSD of the top-ranked pose (model 1) is approximately 8 Å, while other ranks show medians between 15 and 18 Å. PyDock’s lowest median RMSD (∼13 Å) occurs in the second, third, and fourth models, with other ranks ranging between 16 and 18 Å. LZerD performs the worst on the provided data set, with medians between 13 and 21 Å, not identifying the first models as the best ones.

Similarly, the average CIRNet prediction score for core interacting pairs does not show a strong correlation with RMSD, as indicated by a Pearson correlation of −0.14 for ClusPro (*p*-value at 0.05) and LZerD (*p*-value at 0.04), and −0.03 for PyDock (*p*-value at 0.64). To assess if CIRNet can still improve docking pose selection, we analyzed the impact of removing poses not recognized as core interacting residues by CIRNet.

Figure 5b shows the ROC AUC between the RMSD distributions of poses associated with core interacting residues and those removed for various NN prediction thresholds. For each NN cutoff, we computed the average of the AUC obtained for all three servers. A threshold of 0.4 on the NN-predicted binding propensity proves most effective at identifying poorer poses, as indicated by a mean ROC AUC of 0.67. Higher cut-offs do not result in significant improvement in performance.

The bottom plot in Figure 5b details the average RMSD of removed (red) versus selected (green) poses according to this threshold: the former tends to have a higher average RMSD. For ClusPro, 40–50% of poses are removed, with significant RMSD reductions for ranks two, three, seven, and nine (8, 7, 12, and 25%, respectively). PyDock shows similar removal rates (20–50%), with RMSD reductions of about 10, 15, 19, 10, and 23% for ranks one and three through six. LZerD removes fewer poses, conserving around 70% of structures in the top four ranks, but achieves the most significant RMSD improvements (20–30%) for the second, third, and fourth models.

Among the “Docking data set” complexes, 13 lack core interacting residues in their native poses. We included this class of complexes to test CIRNet’s applicability across different dimer types. Figure 5c shows that when only the other 17 complexes are considered, a better identification of poorer poses is achieved, as shown by higher values of the ROC AUC between the RMSD distributions of removed and selected poses. The optimal NN prediction threshold remains 0.4, corresponding to a ROC AUC of approximately 0.8. Using this threshold, similar percentages of poses are removed as before, but with substantially reduced average RMSD. ClusPro’s first two ranks see reductions of approximately 39 and 25%, respectively. PyDock’s reductions are between 15 and 23% for the top three ranks, with a 62% reduction for the fourth rank. LZerD shows reductions of about 10, 30, 58, and 36% for ranks one through four, respectively.

CIRNet performs optimally for complexes with closely positioned core interacting residues and refines docking pose selection even in dimers where the native poses lack core interacting residues. By identifying residue pairs that cannot form the central interaction patches but are incorrectly predicted as such by docking algorithms, CIRNet can effectively filter out the least accurate predictions.

Finally, we compared CIRNet’s classification performance with that of another recently developed tool for docking pose rescaling, TRScore.^25^ Introduced in 2022 by Guo et al., TRScore is a deep learning-based method evaluated on several data sets, including the protein–protein docking benchmark 5.0 update set,^74^ the DockGround decoy set,^42^ and a realistic CAPRI decoy set.^38−40^ Leveraging its deep convolutional RepVGG architecture,^14^ TRScore achieved significant improvements over four state-of-the-art protein–protein docking scoring functions: DOVE,^76^ ZRANK,^58^ ZRANK2,^59^ and IRAD.^73^

Since TRScore was designed to rescale poses, whereas CIRNet was developed to filter out the worst models (resulting in more conservative score assignments), we excluded models with a CIRNet score of zero for a fair comparison. This decision is supported by the observation that a null score is typically assigned to the poorest poses. Across the three docking tools considered, an average of three models per complex are assigned a null score by CIRNet, and their mean RMSD is approximately six times higher than the average RMSD of the remaining poses.

**Figure 6:**
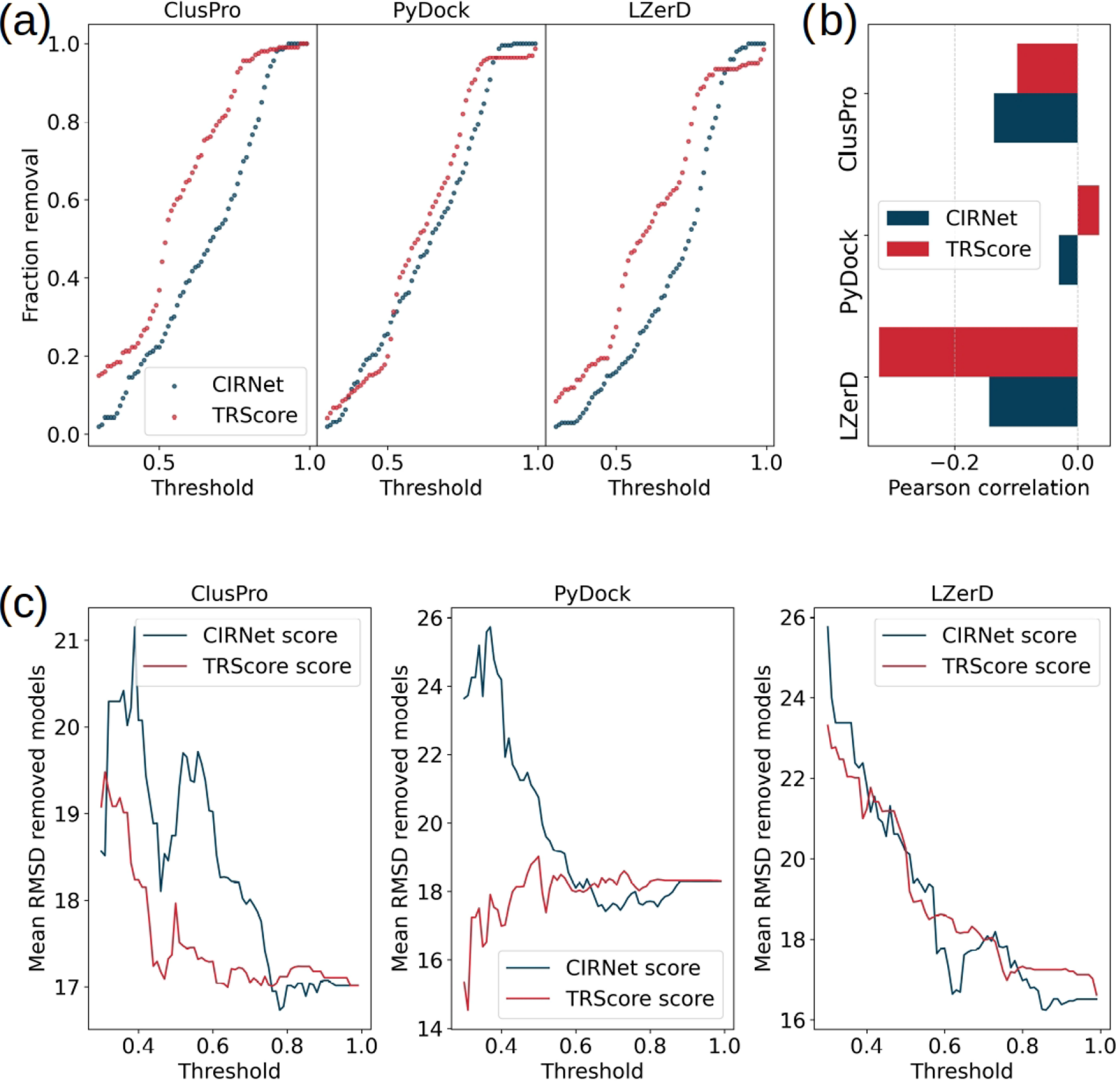
Comparison between CIRNet and TRScore performance. (a) Poses with scores below a given threshold are classified as bad models and removed. The plot illustrates the fraction of poses removed by CIRNet (blue) and TRScore (red) as a function of increasing thresholds. From top to bottom, the models correspond to those generated by the ClusPro, PyDock, and LZerD docking servers. (b) Pearson correlation between the RMSD of each model and the score assigned by CIRNet (blue) and TRScore (red). From top to bottom, the correlation is computed for the docked poses from ClusPro, PyDock, and LZerD. (c) Mean RMSD of the removed poses as a function of increasing thresholds applied to the predictions of CIRNet (blue) and TRScore (red). From left to right, the analysis refers to the models generated by ClusPro, PyDock, and LZerD.

To assess the relative performance of CIRNet and TRScore, we applied TRScore to the “Docking data set”, assigning a score to each model, ranging from zero (worst) to one (best). The scores assigned by both protocols follow a similar distribution. As shown in Figure 6a, the fraction of models with scores below a given threshold exhibits a comparable trend for increasing threshold values for both CIRNet and TRScore.

We then analyzed the rescaling efficiency of the two protocols by computing the Pearson correlation coefficient between scores and RMSD. As shown in Figure 6b, CIRNet demonstrates superior performance compared to TRScore when applied to the poses generated by PyDock and ClusPro, achieving correlations of −0.03 versus 0.03 and −0.17 versus −0.1, respectively. Conversely, TRScore achieves better rescaling results for LZerD, as evidenced by a correlation of −0.32, compared to the −0.14 achieved by CIRNet for this docking server.

Figure 6c shows that a similar trend is observed when evaluating the ability of CIRNet and TRScore to eliminate the worst poses, rather than rescaling all the proposed models. For ClusPro, CIRNet removes models with higher mean RMSD compared to those eliminated by TRScore, up to a threshold value of approximately 0.75 (corresponding to the removal of 60% of the proposed complexes). A similar observation holds for PyDock, where CIRNet outperforms TRScore up to a threshold of approximately 0.6 (resulting in the removal of 40% of the models). Beyond these thresholds, corresponding to the removal of a higher fraction of models, the mean RMSD of the removed poses becomes comparable for both protocols. In contrast, for LZerD, the two protocols perform similarly, removing poses with average RMSD values that do not show significant variation between CIRNet and TRScore scoring.

In conclusion, CIRNet not only matches but often outperforms TRScore in eliminating models far from the native pose. Additionally, for two of the three docking tools considered, CIRNet demonstrates superior performance in rescaling the proposed models.

## 3 Conclusions

Understanding the molecular mechanisms underlying protein binding is a fundamental pursuit in molecular biology, with implications in many applied fields, including protein design and the mapping of the human interactome.^26,35,54,83^ Many of the models used to investigate aggregate-related pathologies indicate dimer formation as the first step involved in protein aggregation,^82^ highlighting the importance of this investigation even for therapeutical applications. A critical aspect of this endeavor is the accurate characterization and prediction of interacting regions.

The properties of the environment, such as pH and ionic strength, as well as the presence of ordered water molecules, and the complexity of protein–protein interactions make the complete characterization of binding sites a demanding problem. Docking servers are instrumental in predicting the most likely poses of protein complexes, yet their performance is often limited by scoring functions that lack sufficient accuracy. Consequently, these scores frequently do not reflect how closely the proposed complex approximates the native pose.

In 2024, Abramson et al. introduced AlphaFold3.^1^ Leveraging a diffusion-based architecture, AlphaFold3 surpassed previous docking tools in accuracy.^1,17,79^ Nevertheless, it has some limitations, for example in predicting the effects of single mutations. This highlights the ongoing need for targeted structural prediction methods.^43^ To address these challenges, we propose a computational strategy that leverages a unified formalism based on the 2D Zernike orthogonal polynomial basis to describe the molecular and electrostatic potential surfaces of proteins. Lennard–Jones and Coulomb potentials, which represent short-range van der Waals forces and long-range electrostatic forces respectively, play crucial roles in protein interactions. These interactions are vital for both the initial recognition of binding partners and subsequent binding adaptation.

Our analysis reveals that interfaces exhibit a core region with a radius of approximately 10 Å where shape complementarity is maximized. This core region also shows higher hydrophobicity complementarity than the rest of the surface.

We combine information on these three aspects to train CIRNet, a neural network designed to identify pairs of interacting residues situated at the core of binding sites. On a balanced data set, the proposed NN achieves a performance of approximately 0.87 in identifying pairs of core interacting residues. In a blind search, CIRNet distinguishes core interacting pairs (residues within 3 and 5 Å from the interface centroid) from negative examples, achieving a ROC AUC of 0.72.

Furthermore, we tested CIRNet as a reranking method for poses proposed by three publicly available docking servers: ClusPro, PyDock, and LZerD. Our results show that the average NN predictions for core interacting pairs tend to be lower (indicating a lower probability of interaction) for poses that are far from the native structure (high RMSD). By removing poses where core interacting residues are not recognized as such by CIRNet, we were able to partially filter out structures that, despite high ranking, are far from the native pose. The best results were achieved for complexes where the native state features closely interacting core residues, but CIRNet’s protocol can be extended to other types of complexes. Even when true binding does not rely solely on core interacting residues, CIRNet can identify pairs that do not meet the physical and geometrical criteria for core binding regions.

In comparison to TRScore, a state-of-the-art tool for rescaling docking poses, CIRNet demonstrated better or comparable performance in removing the worst docking poses. When evaluated as a rescaling tool, CIRNet showed stronger performance, resulting in a better correlation between the predicted score and RMSD for two out of the three docking servers considered in the evaluation. This suggests that CIRNet may offer an improved method for accurately predicting docking pose quality.

The presented strategy is based on a compact and efficient formalism for characterizing portions of the molecular surface of protein structures, as shown in the comparative analyses between interacting and noninteracting regions, thus allowing the use of representative descriptors as inputs for neural networks. The results of this integrated approach, where multiple physicochemical properties are used in a compact and low-approximation manner, highlight CIRNet’s remarkable ability to improve the prediction of protein–protein complexes.

## 4 Methods

### 4.1 “CIRNet Data Set”

To probe the physical and chemical features of protein–protein binding regions, we collected a data set of protein–protein dimers with structural information obtained through X-ray crystallography, sourced from the 3D Complex Database.^41^ Starting from the data set already used by Milanetti et al.,^44^ we selected only pair interactions with known experimental pH values, resulting in the so-called “CIRNet data set”, counting 3721 complexes. These dimers can be classified into four groups based on the chains composition and spatial orientation:

- 614 homodimers with Identical Binding Regions (IBR-hom): binding regions with at least 70% residues overlap.
- 998 homodimers with Shifted Binding Regions (SBR-hom): interacting patches with 30 to 70% of common residues.
- 118 homodimers with non-Identical Binding Regions (nIBR-hom): binding regions sharing less than 30% of residues.
- 1991 heterodimers (Het): complexes involving interactions between two different proteins.

We then removed 100 complexes randomly extracted from the “CIRNet data set”, leaving 3621 for identifying the optimal parameters of a NN to pinpoint core interacting residues and to test its performance on a balanced data set. The resulting architecture was applied in a blind search over the 100 removed complexes. The composition of this subset reflects that of the original data set. Among the 100 complexes, 69 are heterodimers and 31 homodimers. In particular there are 15 SBR-hom, 13 IBR-hom and 3 nIBR-hom.

Of these, 30 complexes were randomly selected and docked using three different docking software: ClusPro, PyDock, and LZerD. These complexes form the so-called “Docking data set”.

It is noteworthy that approximately 60% of the complexes exhibit core interacting residues at their interfaces. We included complexes lacking this characteristic in the data set to test CIRNet’s applicability across different types of complexes. This allowed us to assess CIRNet’s performance in identifying core residues and to use these noncore complexes as negative examples or to evaluate CIRNet’s ability to exclude residue pairs that cannot be at the core of the interfaces.

### 4.2 Computation of the Surfaces and Binding Sites Definition

For each protein in the “CIRNet data set”, the solvent-accessible surface was computed using DMS,^64^ with a resolution of 5 points per Å^2^ and a water probe radius of 1.4 Å. The electrostatic potential was calculated through the Poisson–Boltzmann equation with the APBS code,^32^ taking into account the experimental pH and treating each protein independently from its partner. To define the electrostatic potential surface, we created a grid and assigned electrostatic potential values to each grid cell corresponding to surface points. Binding sites were defined as regions on a protein surface within 6 Å of the partner surface. The center of an interface was determined as the centroid of the surface points within this binding region.

### 4.3 Hydrophobicity Scale

To measure hydropathy, we use the scale introduced by Di Rienzo et al.^11^ to compactly represent and compare the hydrophobicity of molecular surface patches. They conducted MD simulations for each of the 20 natural amino acids and analyzed the variations in the hydrogen bond network of surrounding water molecules. Observing the spatial reorganization in the local structure allowed them to evaluate their hydrophilicity and hydrophobicity features and associate each amino acid with a hydrophobicity index (*H*). The resulting hydrophobicity scale considers the collective response of protein hydration waters to the local nanoscale chemical and topographical patterns.

### 4.4 Patch Definition and Projection

A surface patch is defined by placing a sphere with a radius of *R* = 9 Å centered on a surface point and selecting the surface points within this sphere. Previous studies^21,44^ identified that this range of *R* values is optimal for accurately identifying binding regions based on shape and electrostatic complementarity.

Next, we fit a plane to the selected patch points and reorient the patch so that its normal vector is perpendicular to the plane. To compare shape complementarity between two patches, they must be reoriented so that one patch has its solvent-exposed side facing the positive *z*-axis and the other facing the negative *z*-axis.

Each point on the reoriented patch is described by its three spatial coordinates and the electrostatic potential at that point. These descriptions are then projected onto the *x*–*y* plane.

We define a point *C* on the *z*-axis and set it such that the angle θ between the *z*-axis and any secant connecting C to a point on the patch is θ = 45°. To obtain the shape projection, each surface point is labeled with its distance *r* from C. A 25 × 25 pixels grid is constructed, and each pixel is associated with the mean *r* value calculated on the points projected inside it.

For the electrostatic projection, a separate 25 × 25 grid is created, and each pixel is assigned the mean electrostatic potential of the points projected onto it.

The parameters used for these projections were identified as the most effective in the previously mentioned work.^44^

### 4.5 Zernike 2D Protocol

Any function of two variables *f*(*r*, ψ) defined in polar coordinates inside the region of the unitary circle (in our case shape or electrostatic projection of a surface patch) can be decomposed in the Zernike basis
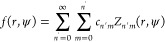
2where
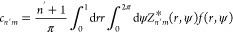
3and
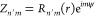
4*c*_*n*′*m*_ are the expansion coefficients, while the complex functions *Z*_*n*′*m*_(*r*, ψ) are the Zernike polynomials. The radial part *R*_*n*′*m*_ is given by
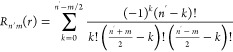
5

The complete set of Zernike polynomials forms a basis for expanding functions on the unit circle. The set of complex expansion coefficients *c*_*n*′*m*_ uniquely reconstructs the original function, with the resolution of this reconstruction dependent on the order of expansion *N* = *max*(*n*′). The norms of these expansion coefficients are known as Zernike invariant descriptors, which are invariant to rotations around the origin of the unit circle.

To quantify the similarity between two functions in polar coordinates, we compute the Euclidean distance between the corresponding Zernike invariant vectors. For our shape- and electrostatic projections, a smaller distance between the Zernike vectors of two patches indicates higher complementarity (note that one of the patches is inverted in this comparison).

In this study, we used *R* = 9 Å and *N* = 20, identified as the most effective parameters in previous work.^44^ Smaller values of *R* would result in patches that are too small to adequately distinguish interactions between regions. Conversely, a larger radius would encompass noninteracting regions that inherently have low complementarity. The order of expansion *N* affects the level of detail captured: lower values lead to excessively “smooth” reconstructions, while excessively high *N* values capture unnecessary details, which can be time-consuming.

### 4.6 Neural Network Architecture

We employ the neural network architecture illustrated in Figure 3b. CIRNet consists of two convolutional layers^36^ followed by two dense layers. To prevent overfitting, the convolutional layers are followed by a dropout layer for regularization. The learned features are then flattened into a 1D vector and passed through two fully connected layers. The final output layer predicts the probability that the input residue pair is a core interacting pair.

For each residue pair, we feed to CIRNet a 4 × 10 matrix that represents the normalized complementarity of the shape, electrostatic, and hydropathy complementarity between the first residue and a 10 Å radius region centered on the second amino acid.

#### 4.6.1 Training and Hyperparameters

After selecting 100 complexes for a final blind test, the remaining protein dimers were randomly divided into training (70%) and testing (30%) sets. For each protein, a patch was centered on every tenth point on the real interface, and the Zernike formalism was applied to these regions. To ensure a balanced set, an equal number of patches were created from points randomly selected outside the binding regions.

For each residue pair, we computed the mean values of the *Z*_s_ (shape complementarity) and *Z*_el_ (electrostatic complementarity) between the patches centered on the respective amino acids. The *H*_*r*_ (hydropathy complementarity) value was also calculated for each pair. Consequently, each residue pair was associated with a matrix and a binary label: 1 if the residues are within 3 Å from each other and less than 5 Å from the interface center; otherwise, 0. The NN was optimized through an Adam version of the stochastic gradient descent.^34^ The training objective was to minimize the binary cross-entropy loss function, comparing the final output prediction to the target label.

#### 4.6.2 Blind Search for Core Interacting Residues

To evaluate the accuracy of the NN in a blind search for core interacting residues, we used the 100 complexes that were excluded from the network’s training and testing data sets. For this assessment, we sampled the entire surface of each protein, defining a patch for every set of 10 points. Subsequently, all possible residue pairs were classified by the NN.

## Data Availability

All relevant data are displayed within the manuscript. Raw data can be requested to the corresponding authors. Codes to reproduce the results are available on: https://github.com/matmi8/Zernike2D
